# Feasibility and First Results of Heart Failure Monitoring Using the Wearable Cardioverter–Defibrillator in Newly Diagnosed Heart Failure with Reduced Ejection Fraction

**DOI:** 10.3390/s21237798

**Published:** 2021-11-23

**Authors:** Henrike Aenne Katrin Hillmann, Stephan Hohmann, Johanna Mueller-Leisse, Christos Zormpas, Jörg Eiringhaus, Johann Bauersachs, Christian Veltmann, David Duncker

**Affiliations:** Hannover Heart Rhythm Center, Department of Cardiology and Angiology, Hannover Medical School, 30625 Hannover, Germany; hillmann.henrike@mh-hannover.de (H.A.K.H.); hohmann.stephan@mh-hannover.de (S.H.); mueller-leisse.johanna@mh-hannover.de (J.M.-L.); zormpas.christos@mh-hannover.de (C.Z.); eiringhaus.joerg@mh-hannover.de (J.E.); bauersachs.johann@mh-hannover.de (J.B.); veltmann.christian@mh-hannover.de (C.V.)

**Keywords:** wearable cardioverter–defibrillator, sudden cardiac death, heart failure, heart failure monitoring

## Abstract

The wearable cardioverter–defibrillator (WCD) is used in patients with newly diagnosed heart failure and reduced ejection fraction (HFrEF). In addition to arrhythmic events, the WCD provides near-continuous telemetric heart failure monitoring. The purpose of this study was to evaluate the clinical relevance of additionally recorded parameters, such as heart rate or step count. We included patients with newly diagnosed HFrEF prescribed with a WCD. Via the WCD, step count and heart rate were acquired, and an approximate for heart rate variability (HRV5) was calculated. Multivariate analysis was performed to analyze predictors for an improvement in left ventricular ejection fraction (LVEF). Two hundred and seventy-six patients (31.9% female) were included. Mean LVEF was 25.3 ± 8.5%. Between the first and last seven days of usage, median heart rate fell significantly (*p* < 0.001), while median step count and HRV5 significantly increased (*p* < 0.001). In a multivariate analysis, a delta of HRV5 > 23 ms was an independent predictor for LVEF improvement of ≥10% between prescription and 3-month follow-up. Patients with newly diagnosed HFrEF showed significant changes in heart rate, step count, and HRV5 between the beginning and end of WCD prescription time. HRV5 was an independent predictor for LVEF improvement and could serve as an early indicator of treatment response.

## 1. Introduction

Patients with heart failure with reduced ejection fraction (HFrEF) secondary to ischemic or non-ischemic cardiomyopathy may be at high risk for malignant arrhythmias and sudden cardiac death [1,2,3] without meeting implantable cardioverter–defibrillator (ICD) implantation criteria [4]. The wearable cardioverter–defibrillator (WCD) can be used in such patients during HFrEF therapy optimization and ongoing risk stratification [2,3,4,5,6]. Previous studies have analyzed the effectiveness of the WCD regarding different indications in patients with ischemic and non-ischemic cardiomyopathy [1,3,7,8,9,10,11,12,13,14]. While the primary purpose of the WCD is the prevention of sudden arrhythmic cardiac death, it also provides near-continuous telemetric monitoring of parameters of interest in heart failure [15,16]. These parameters, such as heart rate or step count, are accessible via a secure web platform (ZOLL Patient Management Network, ZOLL, Pittsburgh, PA, USA).

In clinical practice, patients with newly diagnosed HFrEF are typically discharged on starting doses of guideline-directed medical therapy and are scheduled for a follow-up visit after three months. However, in a significant fraction of patients, medication is not up-titrated to adequate target doses after the first three months [17]. An early identification of patients without sufficient left ventricular ejection fraction (LVEF) improvement would provide the opportunity for intensified follow-up and re-evaluation in this subgroup.

The aim of this study was to evaluate the clinical relevance of the additional telemetric parameters recorded and provided by the WCD and to identify potential early predictors of LVEF improvement at 3-month follow-up.

## 2. Methods

The PROLONG-II study enrolled patients who received a WCD (LifeVest, ZOLL, Pittsburgh, PA, USA) at Hannover Medical School [18]. The aim of the present study was to provide extended follow-up after WCD prescription. A subgroup of patients included in the PROLONG-II study with newly diagnosed heart failure who received a WCD at Hannover Medical School between April 2013 and December 2017 was included in this retrospective single-center analysis. Additional inclusion criteria were an average WCD wear time of at least 20 h per day and the availability of data for at least one additional heart failure parameter of interest (heart rate, step count, or body position). Exclusion criteria were chronic heart failure, e.g. patients after explant of an ICD, an average WCD wear time lower than twenty hours per day, and no available heart failure parameters for heart rate, step count, or body position. 

Baseline characteristics were taken at the date of WCD prescription, at the 3-month follow-up, and date of the latest follow-up, as available. Characteristics included medical history, clinical status, LVEF, and 12-lead ECG. LVEF was routinely assessed by echocardiography using Simpson’s method. All measurements were made in the same center by trained physicians, according to current recommendations for echocardiographic measurements [19,20]. 

All patients gave informed consent. The present study was conducted in compliance with the Declaration of Helsinki and was approved by the Ethics Committee of Hannover Medical School, Germany.

### 2.1. WCD Data

**Figure 1:**
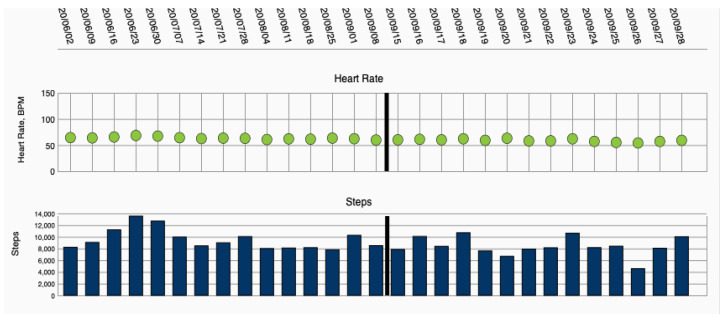
Heart failure parameters heart rate and step count, displayed via remote monitoring.

WCD data were collected via remote monitoring, using an internet platform provided by the manufacturer (ZOLL Patient Management Network, ZOLL, Pittsburgh, PA, USA) (Figure 1).

Heart failure parameters included in the analysis were heart rate and step count, recorded in five-minute intervals. Data were monitored during the period of heart failure therapy optimization. Five-minute heart rate variability approximate (HRV5), a surrogate for beat-to-beat heart rate variability, was calculated as the cycle length standard deviation over all data points per 24-h period. HRV5 is closely related to the standard deviation of the average normal–normal intervals (SDANN), an established measurement of heart rate variability [21]. 

When comparing the first and last seven days of usage, only patients with at least 14 days of wear time were included to avoid data overlap. Using the term “wear time”, following the specific criteria chosen by the WCD manufacturer, the first and last day of wear, as well as days with usage below fifteen minutes per day, were excluded. 

Regarding LVEF response, patients with an absolute improvement of at least 10 percentage points between prescription and 3-month follow-up were classified as LVEF improvers.

### 2.2. Statistics

Data extraction and merging were performed using R (Version 4.0.1, The R Foundation for Statistical Computing, Vienna, Austria) and Python (NumPy, version 1.19.5, and Pandas, version 1.2.4, Python Software Foundation, version 3.8.6). Heart rate was calculated as the mean of a day, and step count was given as the sum of the day before further analysis. For heart rate and HRV5, data provided as value “zero” from the WCD were excluded from further analyses. Categorical variables are presented as numbers and percentages. Continuous data are presented as median and interquartile rate (IQR) or mean and standard deviation (SD) where appropriate. Heart rate (bpm) was converted to cycle length (ms) for further analysis. The Wilcoxon test was used for between-group comparisons. For correlation analysis, Spearman’s correlation coefficient was calculated. In order to illustrate the diagnostic ability of parameters, receiver operating characteristic analysis was performed.

Three-day rolling averages of WCD-derived parameters were compared between baseline (day 3) and day 45. Optimum cut-off values for the change in HRV5, daily step count, and daily mean heart rate were identified through receiver operating curves (ROC) and YOUDEN’s J statistic. These parameters, along with other candidate predictors for LVEF improvement, were used as predictors in univariate logistic regression models. Significant predictors from the univariates models were then used as covariates in a multivariate logistic regression model. Statistical data analysis was performed using SPSS (Version 26, IBM, Armonk, NY USA), R, and the pROC package for R [22]. *p*-values < 0.05 were considered statistically significant.

## 3. Results

### 3.1. Patient Characteristics

Two hundred and seventy-six patients between April 2013 and December 2017 met the inclusion criteria for further heart failure parameter evaluation and were included in this analysis. 

Eighty-eight (31.9%) of the patients were female. Mean age was 57.4 ± 15.3 years. Etiology was non-ischemic in 174 patients (63.0%) and ischemic in 102 patients (37.0%). Non-ischemic etiology majorly included patients with dilated cardiomyopathy (*n* = 127), myocarditis (*n* = 22), peripartum cardiomyopathy (*n* = 21), or other origin (*n* = 4). Fifty-eight point seven percent (*n* = 162) of examined patients suffered from arterial hypertension, 22.5% (*n* = 62) from diabetes mellitus, 33.0% (*n* = 91) from dyslipidemia, and 21.7% (*n* = 60) from renal failure. Forty-three patients (15.6%) had a family history of cardiovascular disease. Mean NYHA functional class at prescription was 2.6 ± 0.8. Mean LVEF at prescription was 25.3 ± 8.5%. 

At 3-month follow-up, the mean percentage of the target dose for heart failure medication regarding beta-blocker, Renin-angiotensin system inhibitors, and mineralocorticoid receptor antagonists was higher than at prescription for all named medications. Mean NYHA functional class at the 3-month follow-up was 2.0 ± 0.6, mean LVEF at the 3-month follow-up was 34.1 ± 10.3%. LVEF improvers had a mean delta of 17.7 ± 6.7% between prescription and 3-month follow-up, while LVEF non-improvers had a mean delta of 1.9 ± 4.8%. Patient characteristics at WCD prescription and 3-month follow-up are presented in Table 1.

### 3.2. WCD-Use

**Figure 2:**
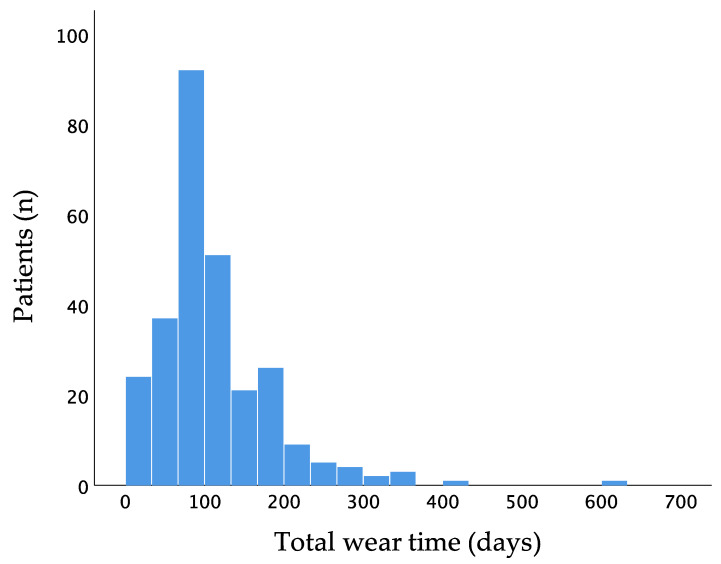
Distribution of wear time of the wearable cardioverter–defibrillator.

Mean wear time of the WCD was 111.8 ± 74.5 days and 23.1 ± 0.9 h per day. Two hundred and sixty-six patients (96.4%) had a wear time of at least 14 days. One hundred and thirty-two patients wore the WCD for at least 90 days (Figure 2).

Sixty-nine patients (25.0%) had a prolonged WCD prescription time after 3-month follow-up. Eleven patients (4.0%) received WCD shocks during the prescription period; all were appropriate shocks for ventricular tachyarrhythmias. There was a total number of 12 delivered WCD shocks. No inappropriate shocks were delivered by the WCD during follow-up. The heart failure etiology was non-ischemic cardiomyopathy in six patients (54.5%) and ischemic cardiomyopathy in five patients (45.5%). Two patients (0.1%) died during the prescription period. One hundred and thirty-three patients (49.6%; *n* = 268) met ICD indication criteria after WCD-prescription time.

Mean change in LVEF between baseline and 3-month follow-up was 9.0 ± 9.7 percentage points, with an LVEF improvement of ≥10 percentage points in 118 patients.

### 3.3. Heart Failure Parameters at Baseline and after Three Months

**Figure 3:**
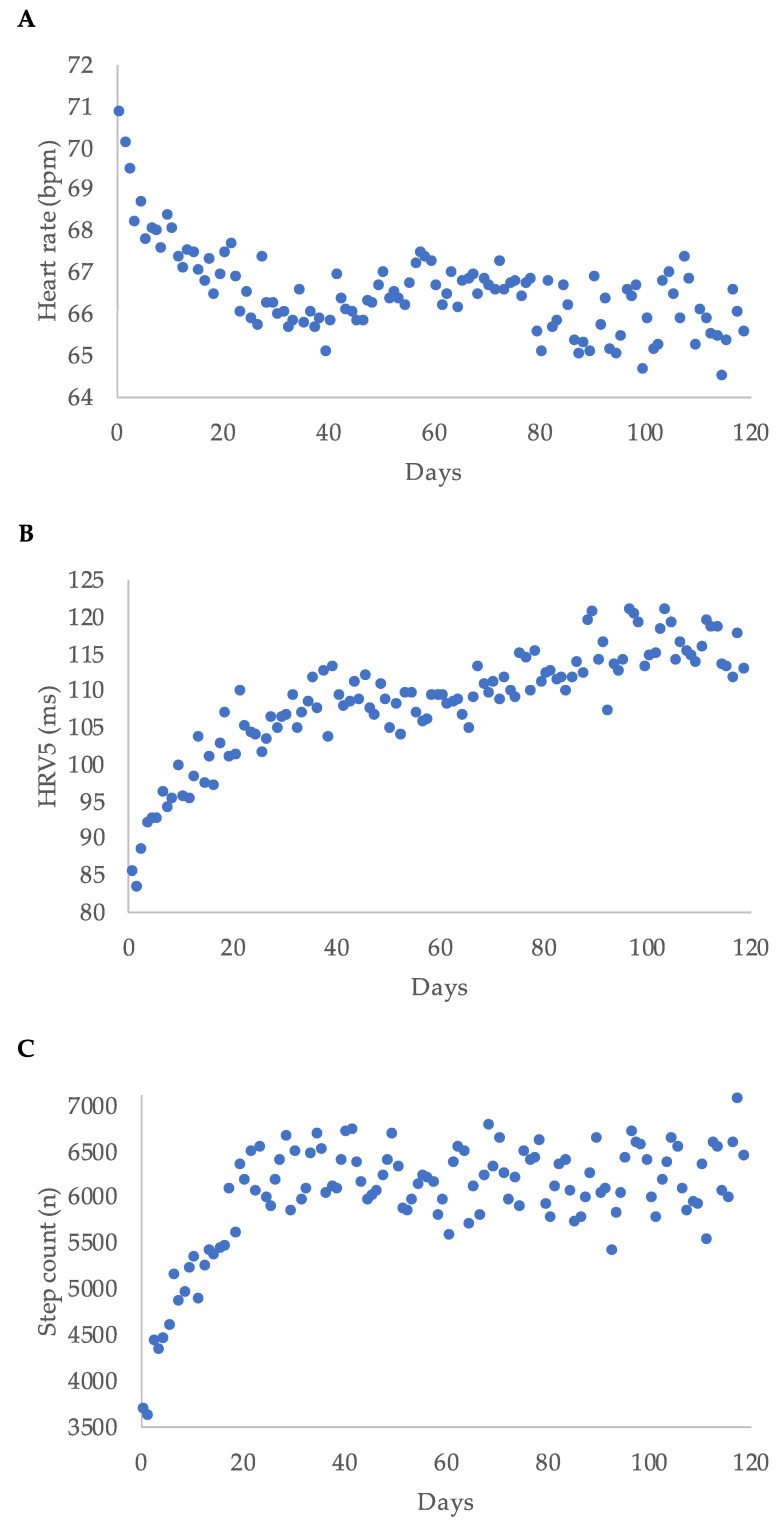
Heart rate (**A**), heart rate variability approximate (HRV5) (**B**), and step count (**C**) displayed as median per day for the first 120 days of wear time for all patients; bpm = beats per minute.

Median heart rate decreased during WCD-wear time, while median step count and median HRV5 increased over time. Regarding the first 120 days of wear-time, the highest delta was within the first 20 days for each parameter (Figure 3).

**Figure 4:**
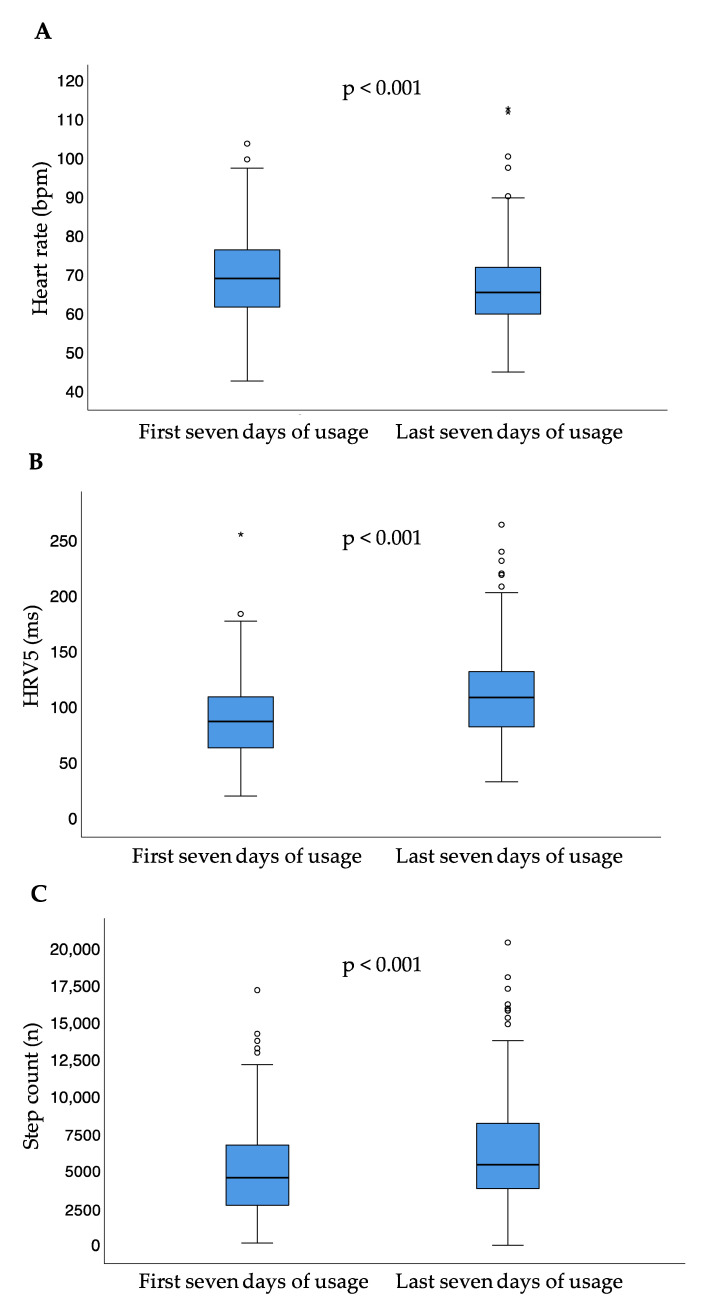
Heart rate (**A**), heart rate variability approximate (**B**), and step count (**C**) for the first and last seven days of usage; small circles represent outliers (data points outside the range of 3rd quartile + 1.5* interquartile range or 1st quartile − 1.5* interquartile range); asterisk represent extreme outliers (data points outside the range of 3rd quartile + 3* interquartile range or 1st quartile − 3* interquartile range); bpm = beats per minute; HRV5 = heart rate variability approximate.

Between the first and last seven days of usage, median heart rate significantly decreased (*p* < 0.001), while median step count per day (*p* < 0.001) and HRV5 significantly increased (*p* < 0.001) (Table 2, Figure 4).

**Figure 5:**
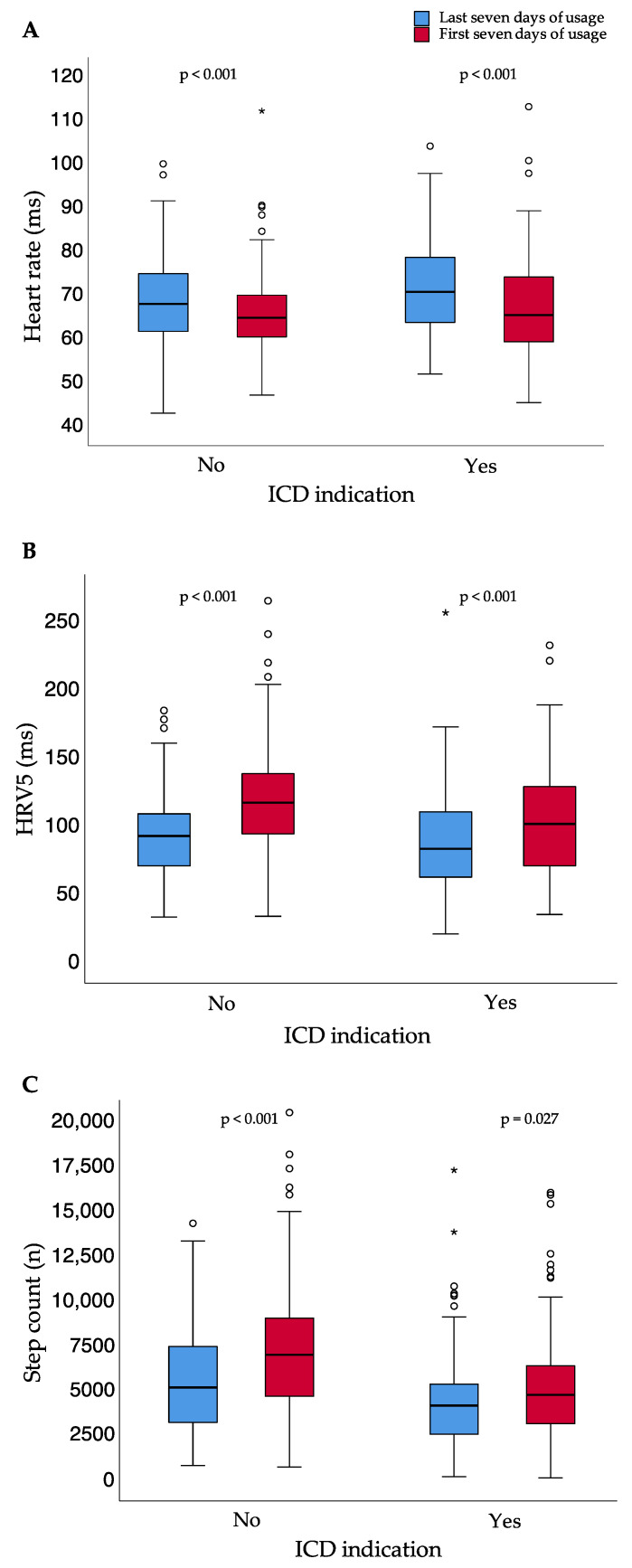
Heart rate (**A**), heart rate variability approximate (**B**), and step count (**C**) for the first and last seven days of usage for patients with and without ICD indication after the end of WCD prescription time; small circles represent outliers (data points outside the range of 3rd quartile + 1.5* interquartile range or 1st quartile − 1.5* interquartile range); asterisk represent extreme outliers (data points outside the range of 3rd quartile + 3* interquartile range or 1st quartile − 3* interquartile range); bpm = beats per minute; ICD = implantable cardioverter–defibrillator; HRV5 = heart rate variability approximate.

The results were independent of whether patients did or did not meet the criteria of ICD indication after the prescription time (Figure 5).

Comparing the first and last seven days of usage, the median difference for the heart failure parameter heart rate was 2.8 (IQR −1.7–7.8) bpm. The median difference for HRV5 was 15.6 (−5.4–42.0) ms. The median difference for step count between the first and last seven days of usage was 712.6 (IQR −676.7–2763.6) steps per day. Executed analyses showed a significance in correlation analysis for several parameters, all associated with correlation coefficients < 0.5 points (Table 3).

### 3.4. Early Predictors of LVEF Improvement

As shown in Figure 3, there was a steep change in heart rate, HRV5, and step count within the first couple of weeks in most patients. We hypothesized that the change in these parameters between baseline and day 45 (the difference in daily average heart rate expressed as cycle length, ΔCL; the difference in HRV5, ΔHRV5; and the difference in daily step count, Δsteps) could predict LVEF response after three months. Using receiver operating curves with ΔCL, ΔHRV5, and Δsteps, we identified the best cut-off values for the prediction of an LVEF improvement of at least 10 percentage points (Table 4).

In univariate logistic regression models, an increase in HRV5 of more than 23 ms, an increase in the daily average cycle length of more than 112 ms, and an increase in the daily step count of more than 1163 steps at day 45 were all significant predictors of ≥10% absolute LVEF improvement by three months. Other significant predictors of improvement were female sex, younger age, non-ischemic cardiomyopathy, and a lower baseline LVEF. When using the significant covariates in a multivariate regression, non-ischemic cardiomyopathy, lower baseline LVEF, and HRV5 increase were still significant predictors (Table 5).

## 4. Discussion

The present study evaluated for the first-time heart failure parameters recorded via the WCD in patients with newly diagnosed heart failure. The main findings are:

1. Patients with newly diagnosed HFrEF show a decrease in heart rate, as well as an increase in heart rate variability approximate and step count during the first three months of heart failure treatment.
2. A higher delta of heart rate or step count between the first and last seven days of usage correlates with a higher delta of the heart rate variability approximate.
3. A delta of heart rate variability approximately >23 ms within the first 45 days of WCD wear time was an independent predictor of LVEF improvement.

This study is the first to analyze the parameters heart rate and step count, as provided and continuously monitored by the WCD additionally to SCD prevention. Patients with newly diagnosed HFrEF without meeting ICD implantation criteria were included in this analysis, representing a well-established indication for WCD prescription [16,17,23]. Patient characteristics in this study were comparable to previous studies regarding the use of the WCD [17,24,25,26]. Heart rate and heart rate variability are especially important parameters for heart failure therapy optimization and long-term management of heart failure [19]. Analyzed heart failure parameters were provided in short 5-min intervals, representing a real-time overview during day and night.

### 4.1. Heart Rate

Several population-based cohort studies have shown an association between a higher resting heart rate and cardiovascular mortality [27,28,29,30]. Moreover, a lower resting heart rate is associated with a lower mortality rate in patients suffering from HFrEF, especially in sinus rhythm [31]. In patients with ischemic cardiomyopathy, a higher resting heart rate is a known factor for a severe outcome [32,33] and is associated with the infarct size after myocardial infarction [34]. In patients with dilated idiopathic cardiomyopathy, a higher resting heart rate is associated with a lower LVEF [35] and is a predictor for arrhythmic events [36]. A resting heart rate <70 bpm is recommended in symptomatic patients with HFrEF, a severely reduced LVEF, and sinus rhythm [19]. Online monitoring using the WCD heart rate monitor may support reaching this aim during up-titration of heart failure medication [37].

In this study, patients showed a significant decrease in heart rate, most likely attributed to an early start of heart failure therapy. There is a further increase in heart rate during WCD-wear time after recompensation and during further heart failure therapy optimization. Data showed a positive correlation between the delta of heart rate and HRV5. Nevertheless, there was no significant correlation between the delta of heart rate and step counts per day. Furthermore, there was no significant correlation between heart rate and LVEF.

### 4.2. Heart Rate Variability

Heart rate variability is a noninvasive method to assess the automatic nervous system function and the automatic activity in patients suffering from cardiovascular diseases [21]. A higher heart rate variability suggests a higher parasympathetic activity [21]. Reduced heart rate variability is a known predictor for mortality in patients with ischemic cardiomyopathy after myocardial infarction [21] or non-ischemic cardiomyopathy [38]. Furthermore, it is a predictor for hospitalization in HFrEF patients with cardiac resynchronization therapy [39].

In this study, we calculated an approximate for the heart rate variability using the standard deviation of the cycle lengths of the given five-minute heart rate data, based on the heart rate variability component SDANN.

We found a significant positive correlation between the delta of HRV5 and the delta of step counts per day between the first and last seven days of usage. A higher number of step counts per day suggests a higher amount of exercise per day. Results of this study are in line with previous studies, which have shown a correlation between exercise and heart rate variability [40,41,42]. There was a significant correlation between the delta of HRV5 between the first and last seven days of usage and the delta of LVEF, an important predictor for sudden cardiac death [4], when measured at prescription and three-month follow-up. This effect may be due to an early start of heart failure medication with an effective dosage and a continued heart failure therapy optimization during follow-up. Furthermore, a delta of HRV > 23 ms within the first 45 days of WCD wear time was shown as an independent predictor of LVEF improvement. Measurement of the heart rate variability using the WCD may be a helpful tool for early prediction of recovery and non-recovery and, therefore, as an early indicator of treatment response, important for HFrEF management.

### 4.3. Step Count

Step count monitoring is popular in the general population, using wearable devices such as smartphones, smartwatches, or wearable pedometers [43]. Just as described above for the resting heart rate, population-based cohort studies showed that a higher number of steps per day correlated with a lower mortality rate in a cohort of adults [44]. In the MADIT-CRT trial, a reduction in activity level was evaluated as a short-term predictor for adverse cardiovascular events in patients with heart failure and implanted cardiac resynchronization device [45].

The present study showed an increase in step count per day over time, which reflected an improvement in heart failure symptoms after initiation and up-titrating of heart failure medication. Moreover, a higher delta of step count per day between the first and last seven days of usage correlated with a higher delta of the LVEF, when measured at prescription and 3-month follow-up.

### 4.4. Predictors of LVEF Improvement and Clinical Application

In the present study, ischemic cardiomyopathy and a higher LVEF at baseline were shown to be independent predictors for an LVEF improvement of less than 10 points at the 3-month follow-up. In comparison, a delta of HRV5 >23 ms within the first 45 days of wear-time was shown to be an independent predictor for LVEF improvement >10% between prescription and 3-month follow-up. An improvement in LVEF with more than 10 percentage points between the beginning of prescription and three-month follow-up was used as the cut-off for LVEF improvement. The cut-off is comparable to the used cut-off in other studies for identifying responders to heart failure therapy [46]. Furthermore, it has been shown to be an independent predictor for a better long-term outcome in patients with heart failure [47]. Whereas indication and baseline LVEF are known and non-changeable factors at prescription in patients with newly diagnosed heart failure, the heart rate variability is a dynamic parameter.

Via remote monitoring, it may be possible to closely monitor analyzed heart failure parameters in patients prescribed with a WCD. Analyzed parameters, especially heart rate variability, can be used for clinical decision-making during heart failure therapy. This process may improve heart failure management using the WCD.

Patients with a significant change in HRV5 until day 45 may be more likely to be LVEF-improvers at the 3-month follow-up. Furthermore, LVEF improvers without reaching a LVEF > 35% at the 3-month follow-up may benefit especially from prolonged WCD-prescription time. Further, prospective studies are needed to confirm this result. On the other hand, patients without an adequate change regarding heart failure parameters, especially the improvement in HRV5, can be selected early. Lack of HRV5 improvement at 45 days could trigger additional interventions, such as additional in-office visits and more aggressive optimization of medical treatment for HFrEF to increase the possibility of LVEF-improvement and avoid ICD-implantation. In contrast to other remote heart failure monitoring systems [48,49,50], no additional devices are needed to monitor heart failure parameters important for LVEF improvement in patients with HFrEF and prescribed WCD.

Management of heart failure therapy and, therefore, LVEF at the 3-month follow-up may be improved, and the number of patients without a need of an ICD device may increase due to the possibility of closer monitoring of additional heart failure parameters.

### 4.5. Limitations

This study represents a real-world collective with the known limitations of retrospective analyses. In order to aim for robust and complete datasets, only patients with a wear time of at least 20 h per day were included in this study, resulting in a selection bias. Furthermore, heart rate variability data were only provided in 5-min intervals. Thus, we derived HRV5 as an approximate metric of heart rate variability.

## 5. Conclusions

The WCD provides heart failure monitoring using additional parameters. Patients with newly diagnosed HFrEF showed significant changes in heart rate, step count per day, and heart rate variability during WCD prescription. Heart rate variability was an independent predictor for LVEF improvement and could serve as an early indicator of treatment response. Remote monitoring for heart failure parameters might be helpful for close monitoring and further HFrEF therapy optimization during WCD wearing.

## Figures and Tables

**Table 1:** Patient characteristics at WCD prescription and 3-month follow-up; SD = standard deviation; LVEF = left ventricular ejection fraction; WCD = wearable cardioverter–defibrillator.

Patient Characteristics	Baseline (*n* = 276)	3-Month Follow-Up (*n* = 271)
NYHA functional class (mean ± SD)	2.6 ± 0.8	2.0 ± 0.6
LVEF (%; mean ± SD)	25.3 ± 8.5	34.1 ± 10.3
Beta-blocker (*n*, %)	260 (94.2)	255 (94.1)
% target dose (mean ± SD)	51.6 ± 28.0	62.5 ± 29.7
Renin–angiotensin system inhibitor (*n*, %)	264 (95.7)	265 (97.8)
% target dose (mean ± SD)	47.9 ± 28.4	61.8 ± 31.7
Mineralocorticoid receptor antagonist (*n*, %)	233 (84.4)	235 (86.7)
% target dose (mean ± SD)	46.1 ± 23.4	54.2 ± 28.9
Diuretics (*n*, %)	217 (78.6)	213 (78.6)
Digitalis (*n*, %)	25 (9.1)	20 (7.4)
Ivabradine (*n*, %)	57 (20.7)	48 (17.7)

**Table 2:** Median heart rate, step count per day, and HRV5 between the first and last seven days of usage.

Parameter	First Seven Days of Usage	Last Seven Days of Usage	*p*-Value
Heart rate (bpm; median; (IQR))	69.5 (62.0–76.8)	65.9 (60.4–72.2)	<0.001
Step count per day (*n*; median; (IQR))	4657 (2778–6918)	5562 (3890–8446)	<0.001
HRV5 (ms; median; (IQR))	89.0 (64.8–110.7)	111.0 (83.7–134.7)	<0.001

**Table 3:** Correlation analysis: Changes in heart failure parameters between the first and last seven days of usage, and, for left ventricular ejection fraction, between prescription and end of WCD wear time, respectively; WCD = wearable cardioverter–defibrillator; LVEF = left ventricular ejection fraction; HRV5 = heart rate variability approximate.

Correlation Analysis for Heart Failure Parameters	Correlation Coefficient (r)	*p*-Value
∆Heart rate/∆HRV5	0.382	<0.001
∆Heart rate/∆Step count per day	0.068	0.297
∆Step count per day/∆HRV5	0.320	<0.001
Age/∆Step count per day	−0.256	<0.001
Age/∆HRV5	−0.143	0.029
Age/∆Heart rate	0.048	0.465
Age/∆LVEF	−0.251	<0.001
∆LVEF/∆HRV5	0.255	<0.001
∆LVEF/∆Step count per day	0.189	0.005
∆LVEF/∆Heart rate	0.028	0.684

**Table 4:** Area under the receiver operating curve (AUC) and optimal threshold for the change in daily average heart rate expressed as cycle length (ΔCL), the difference in HRV5 (ΔHRV5), and the difference in daily step count (Δsteps) for the prediction of an absolute LVEF improvement of ≥10%; AUC = area under the curve; HRV5 = heart rate variability approximate; CL = cycle length.

Parameter	AUC	Optimal Cut-Off
ΔHRV5	0.678	23 ms
ΔCL	0.566	112 ms
Δsteps	0.625	1163 steps

**Table 5:** Univariate and multivariate logistic regression for an LVEF improvement of at least 10% between prescription and three-month follow-up with the delta of HRV5 and CL measured within the first 45 days of WCD wear time; WCD = wearable cardioverter–defibrillator; LVEF = left ventricular ejection fraction; SD = standard deviation; CL = cycle length; ICM = ischemic cardiomyopathy; HRV5 = heart rate variability approximate. In 10 patients no exact LVEF at three months was available and thus no response could be classified. *p*-values given are for logistic regression models, see text.

Parameters	LVEF Improvement < 10% (*n* = 143)	LVEF Improvement ≥ 10% (*n* = 118)	*p*-Value (uni)	Odds Ratio in Multivariate Analysis (95%-CI)	*p*-Value (Multi)
Female Sex (*n*, %)	39 (27.3%)	47 (39.8%)	0.032	1.02 (0.51–2.05)	0.95
Age (mean ± SD)	60.9 ± 14.3	52.6 ± 15.5	<0.001	0.98 (0.96–1.01) per year	0.130
ICM (*n*, %)	69 (48.3%)	22 (18.6%)	<0.001	0.39 (0.18–0.84)	0.018
Baseline LVEF (%; mean ± SD)	27.0 ± 8.4	22.5 ± 7.1	<0.001	0.92 (0.88–0.96) per percentage point	<0.001
∆HRV5 (ms; mean ± SD)	12.9 ± 39.9	38.8 ± 42.2	<0.001	2.13 (1.05–4.32) for ∆HRV5 > 23 ms	0.035
∆CL (ms; mean ± SD)	28.1 ± 119.0	54.6 ± 154.4	<0.001	1.98 (0.89–4.44) for ∆CL > 112 ms	0.093
∆steps (*n*; mean ± SD)	1470 ± 3322	3287 ± 4057	0.002	1.94 (0.99–3.86) for ∆steps > 1163	0.054

## Data Availability

The data of this study are available from the corresponding author, D.D., upon reasonable request.
